# The genome sequence of the Lunar-spotted Pinion, *Cosmia pyralina *(Denis & Schiffermüller, 1775)

**DOI:** 10.12688/wellcomeopenres.20148.1

**Published:** 2023-11-23

**Authors:** Douglas Boyes, Inez Januszczak, University of Oxford and Wytham Woods Genome Acquisition Lab, Darwin Tree of Life Barcoding collective, Wellcome Sanger Institute Tree of Life programme, Wellcome Sanger Institute Scientific Operations: DNA Pipelines collective, Tree of Life Core Informatics collective, Darwin Tree of Life Consortium

**Affiliations:** 1 UK Centre for Ecology & Hydrology, Wallingford, England, UK; 2 Natural History Museum, London, England, UK

**Keywords:** Cosmia pyralina, Lunar-spotted Pinion, genome sequence, chromosomal, Lepidoptera

## Abstract

We present a genome assembly from an individual male *Cosmia pyralina* (the Lunar-spotted Pinion; Arthropoda; Insecta; Lepidoptera; Noctuidae). The genome sequence is 803.3 megabases in span. Most of the assembly is scaffolded into 31 chromosomal pseudomolecules, including the Z sex chromosome. The mitochondrial genome has also been assembled and is 15.39 kilobases in length. Gene annotation of this assembly on Ensembl identified 19,901 protein coding genes.

## Species taxonomy

Eukaryota; Metazoa; Eumetazoa; Bilateria; Protostomia; Ecdysozoa; Panarthropoda; Arthropoda; Mandibulata; Pancrustacea; Hexapoda; Insecta; Dicondylia; Pterygota; Neoptera; Endopterygota; Amphiesmenoptera; Lepidoptera; Glossata; Neolepidoptera; Heteroneura; Ditrysia; Obtectomera; Noctuoidea; Noctuidae; Ipimorphinae; *Cosmia;* Cosmia pyralina (Denis & Schiffermüller, 1775) (NCBI:txid987909).

## Background

The Lunar-spotted Pinion ( *Cosmia pyralina*) is a noctuid moth, commonly found in central Europe, but its territory extends all the way through the Palaearctic to Korea and Japan ( Fauna Europea, 2023). In Britain this is a species of central and southern England and (albeit more scarcely) Wales, particularly the south-east.

With a wingspan of 28–32 mm, the Lunar-spotted Pinion could be confused with the Lesser-spotted Pinion, *Cosmia affinis* (Linnaeus, 1767), but has paler hindwings and broader forewings ( Waring *et al.*, 2017). *Cosmia pyralina* has a variety of colour morphs, with dull or bright red-brown forewings, with a contrasting white edge in the outer line, that eventually joins the white streak before the submarginal line. Similarly, larvae are pale green with white lines. The larvae are polyphagous and feed on deciduous trees, such as blackthorn ( *Prunus spinosa*) and hawthorn ( *Crataegus monogyna*) as well as English elm ( *Ulmus procera*) and wych elm ( *U. glabra*) ( Waring *et al.*, 2017)

The species is declining in Britain ( Conrad *et al.*, 2006), most likely due to Dutch elm disease. Caused by the fungus *Ophiostoma novo-ulmi*, Dutch elm disease is one of the most severe tree diseases in the world, causing foliage and tip dieback in all of Britain’s major native elms ( Potter *et al.*, 2011). Ten beetle species from the genus *Scotylus* (a genus of bark beetle) are known to feed on elms and a key insect vector for the disease to spread. The beetles frequently visit or inflict wounds in healthy elms; thus, depositing any spores directly into the damaged tree ( Peacock *et al.*, 1981). Other *Cosmia* species which have suffered due to this disease include the White-spotted Pinion, *Cosmia diffinis*, which is now listed as ‘Nationally Scarce’ ( Fox *et al.*, 2019). The fourth British *Cosmia* species, *C. trapezina* (the Dun-bar), still has a healthy population despite the described challenges, perhaps due to its trait of eating the larvae of other moth species as well as cannibalising its own ( Turčáni & Patočka, 2011; Ward, 1903).

We present a chromosomally complete genome sequence for the Lunar-spotted Pinion ( *Cosmia pyralina*) as part of the Darwin Tree of Life Project. This project is a collaborative effort to sequence all named eukaryotic species in the Atlantic Archipelago, encompassing Britain and Ireland.

## Genome sequence report

**Figure 1.**
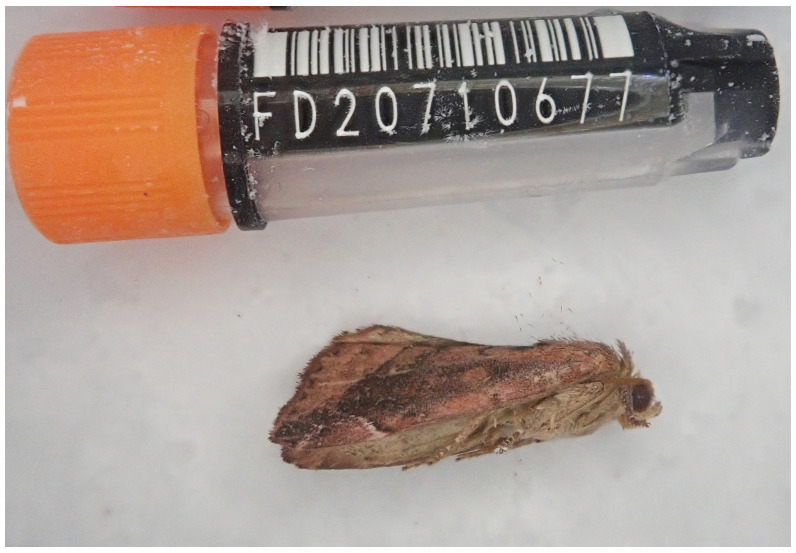
Photograph of the *Cosmia pyralina* (ilCosPyra2) specimen used for genome sequencing.

The genome was sequenced from one male *Cosmia pyralina* ( Figure 1) collected from Wytham Woods, Oxfordshire, UK (51.77, –1.34). A total of 22-fold coverage in Pacific Biosciences single-molecule HiFi long reads was generated. Primary assembly contigs were scaffolded with chromosome conformation Hi-C data. Manual assembly curation corrected 26 missing joins or mis-joins and removed 1 haplotypic duplication, reducing the scaffold number by 18.01% and increasing the scaffold N50 by 1.63%.

**Figure 2.**
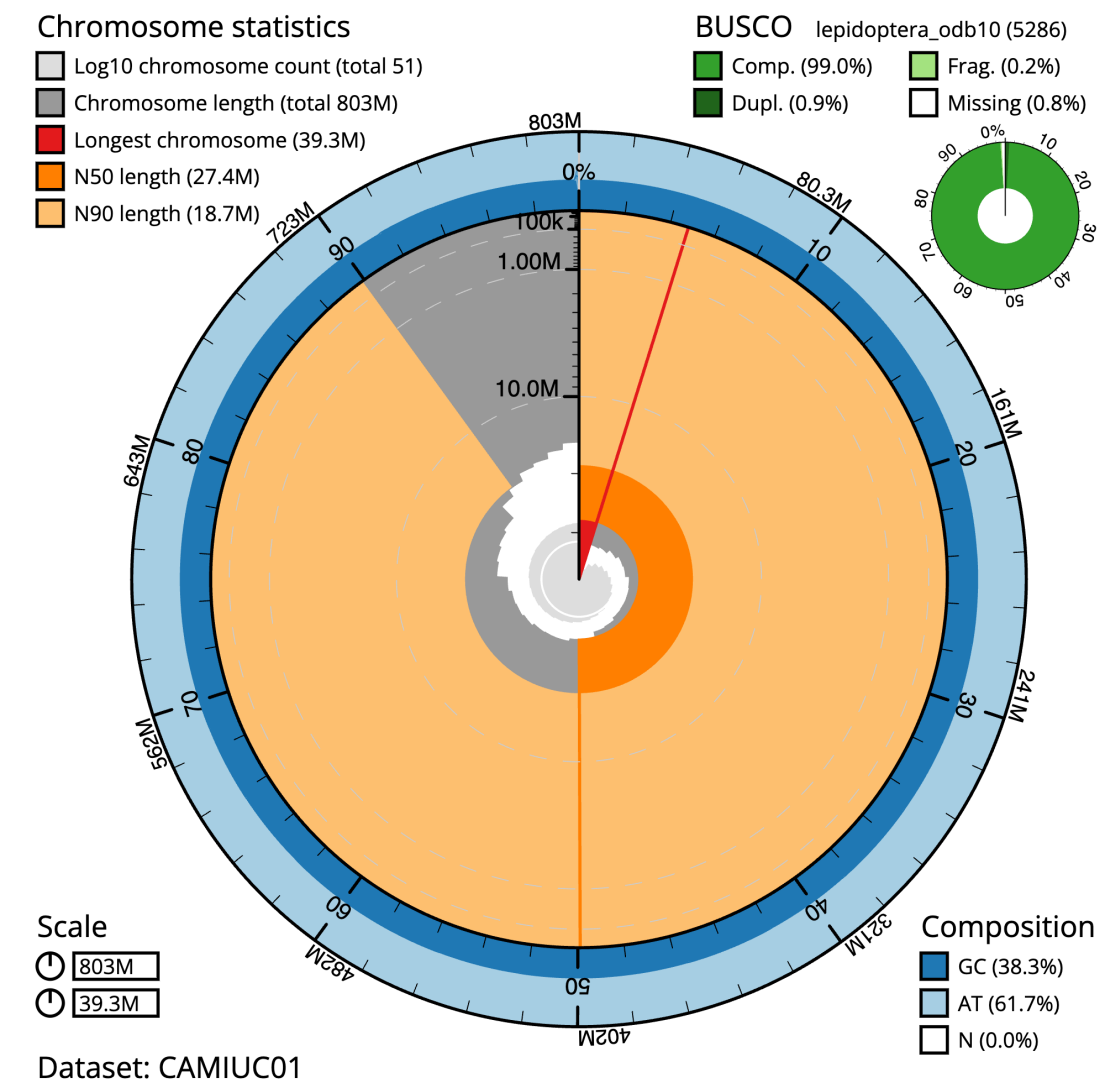
Genome assembly of *Cosmia pyralina*, ilCosPyra2.1: metrics. The BlobToolKit Snailplot shows N50 metrics and BUSCO gene completeness. The main plot is divided into 1,000 size-ordered bins around the circumference with each bin representing 0.1% of the 803,276,861 bp assembly. The distribution of scaffold lengths is shown in dark grey with the plot radius scaled to the longest scaffold present in the assembly (39,281,260 bp, shown in red). Orange and pale-orange arcs show the N50 and N90 scaffold lengths (27,366,473 and 18,734,863 bp), respectively. The pale grey spiral shows the cumulative scaffold count on a log scale with white scale lines showing successive orders of magnitude. The blue and pale-blue area around the outside of the plot shows the distribution of GC, AT and N percentages in the same bins as the inner plot. A summary of complete, fragmented, duplicated and missing BUSCO genes in the lepidoptera_odb10 set is shown in the top right. An interactive version of this figure is available at https://blobtoolkit.genomehubs.org/view/ilCosPyra2.1/dataset/CAMIUC01/snail.

**Figure 3.**
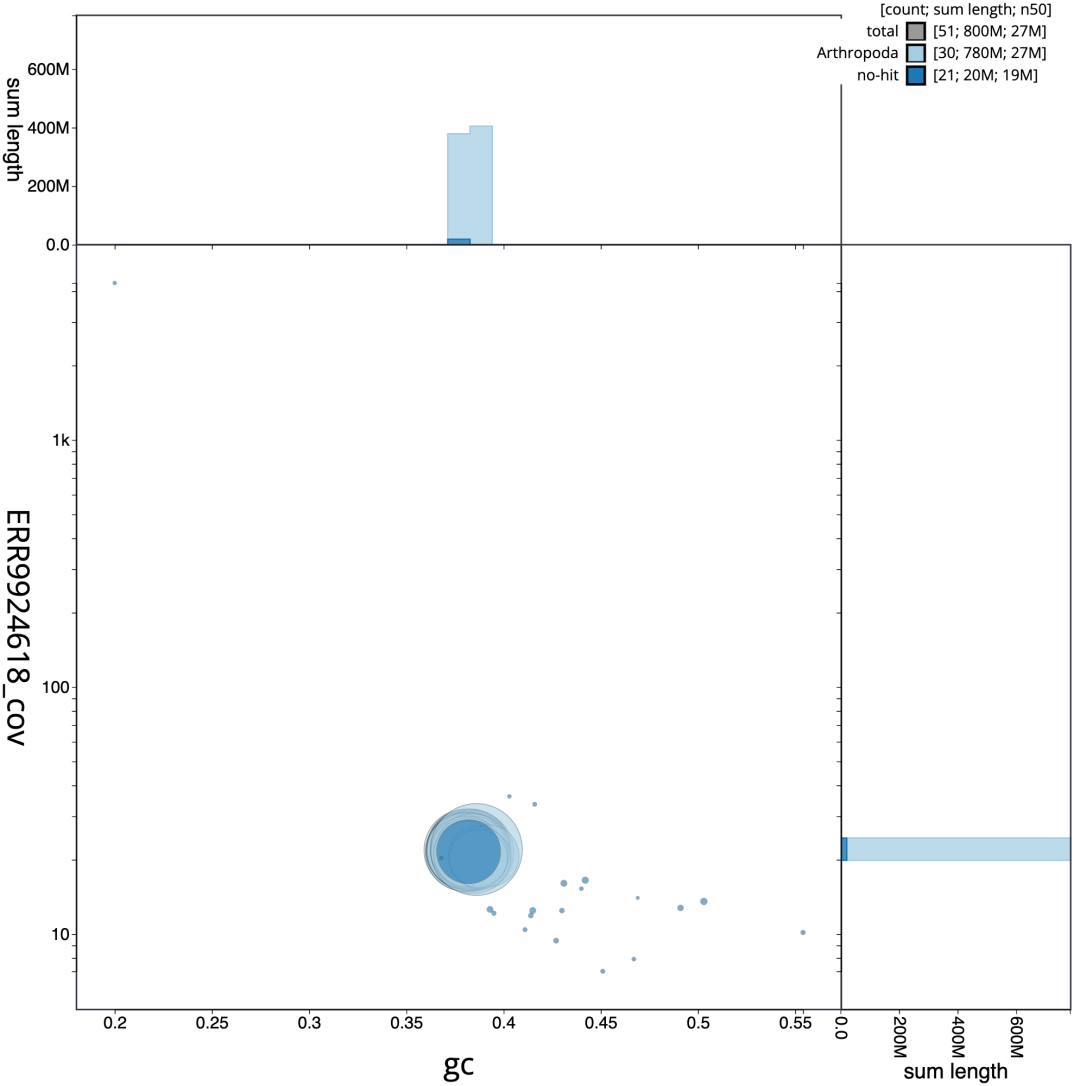
Genome assembly of *Cosmia pyralina*, ilCosPyra2.1: BlobToolKit GC-coverage plot. Scaffolds are coloured by phylum. Circles are sized in proportion to scaffold length. Histograms show the distribution of scaffold length sum along each axis. An interactive version of this figure is available at https://blobtoolkit.genomehubs.org/view/ilCosPyra2.1/dataset/CAMIUC01/blob.

**Figure 4.**
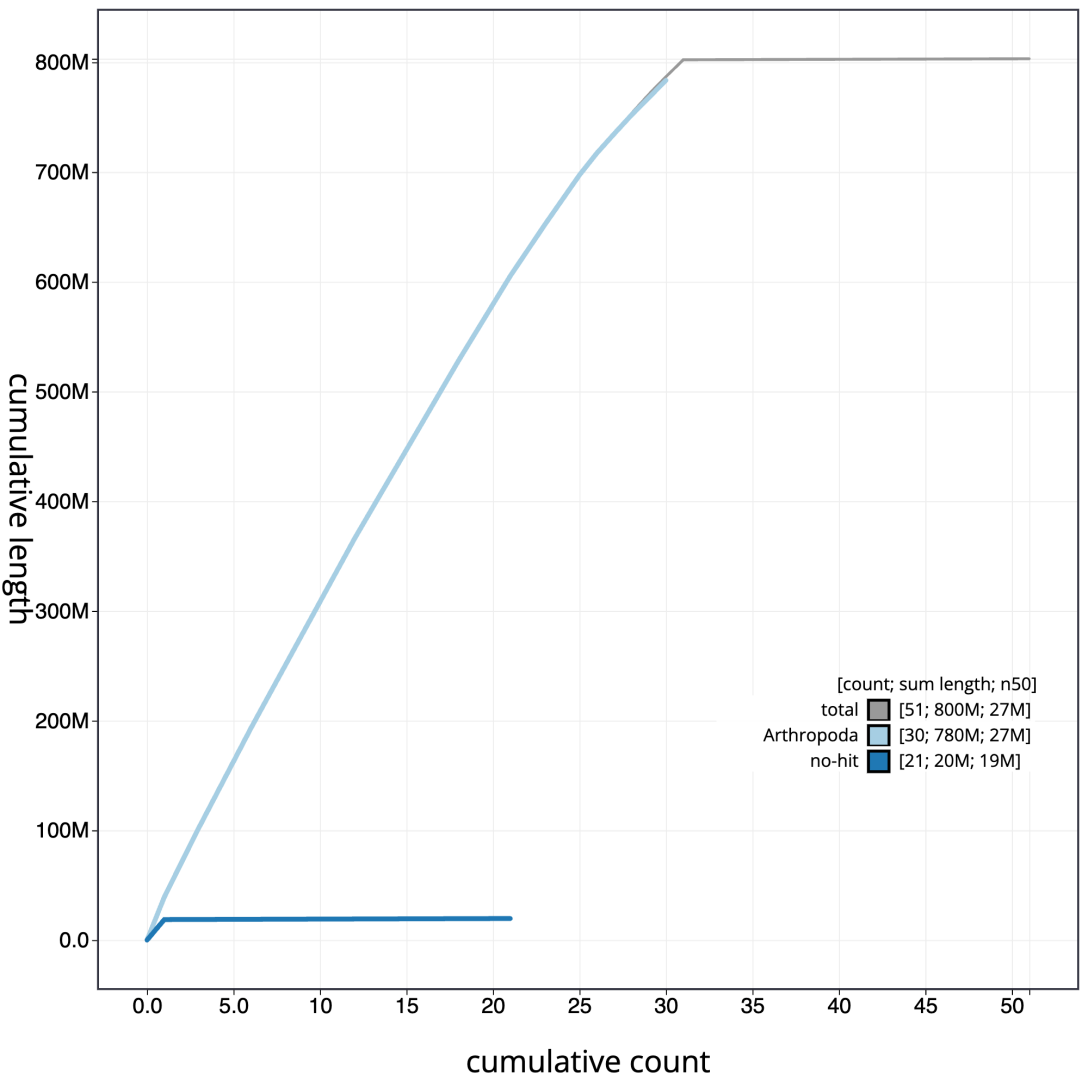
Genome assembly of *Cosmia pyralina*, ilCosPyra2.1: BlobToolKit cumulative sequence plot. The grey line shows cumulative length for all scaffolds. Coloured lines show cumulative lengths of scaffolds assigned to each phylum using the buscogenes taxrule. An interactive version of this figure is available at https://blobtoolkit.genomehubs.org/view/ilCosPyra2.1/dataset/CAMIUC01/cumulative.

**Figure 5.**
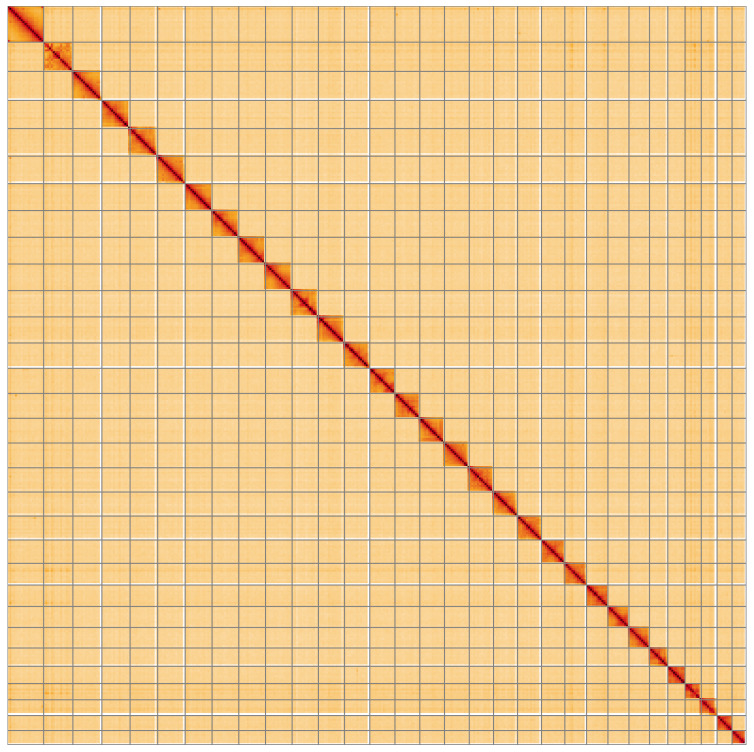
Genome assembly of *Cosmia pyralina*, ilCosPyra2.1: Hi-C contact map of the ilCosPyra2.1 assembly, visualised using HiGlass. Chromosomes are shown in order of size from left to right and top to bottom. An interactive version of this figure may be viewed at https://genome-note-higlass.tol.sanger.ac.uk/l/?d=TpGzUdJFRHyRe3gFUR058w.

The final assembly has a total length of 803.3 Mb in 50 sequence scaffolds with a scaffold N50 of 27.4 Mb ( Table 1). Most (99.89%) of the assembly sequence was assigned to 31 chromosomal-level scaffolds, representing 30 autosomes and the Z sex chromosome. A summary of the assembly statistics is shown in Figure 2, while the distribution of assembly scaffolds on GC proportion and coverage is shown in Figure 3. The cumulative assembly plot in Figure 4 shows curves for subsets of scaffolds assigned to different phyla. Chromosome-scale scaffolds confirmed by the Hi-C data are named in order of size ( Figure 5; Table 2). Comparators *Cosmia trapezina* (GCA_905163495.2) ( Boyes *et al.*, 2022) and *Amphipoea oculea* (GCA_945859645.1) ( Boyes *et al.*, 2023) were used for Z chromosome identification. While not fully phased, the assembly deposited is of one haplotype. Contigs corresponding to the second haplotype have also been deposited. The mitochondrial genome was also assembled and can be found as a contig within the multifasta file of the genome submission.

**Table 1.** Genome data for *Cosmia pyralina*, ilCosPyra2.1.

Project accession data		
Assembly identifier	ilCosPyra2.1	
Species	*Cosmia pyralina*	
Specimen	ilCosPyra2	
NCBI taxonomy ID	987909	
BioProject	PRJEB54090	
BioSample ID	SAMEA10979084	
Isolate information	ilCosPyra2, male: head and thorax (DNA sequencing and Hi-C scaffolding), abdomen (RNA sequencing)	
Assembly metrics *		*Benchmark*
Consensus quality (QV)	61.5	*≥ 50*
*k*-mer completeness	100%	*≥ 95%*
BUSCO **	C:99.0%[S:98.1%,D:0.9%],F:0.2%, M:0.8%,n:5,286	*C ≥ 95%*
Percentage of assembly mapped to chromosomes	99.89%	*≥ 95%*
Sex chromosomes	Z chromosomes	*localised homologous pairs*
Organelles	Mitochondrial genome assembled	*complete single alleles*
Raw data accessions		
PacificBiosciences SEQUEL II	ERR9924618	
Hi-C Illumina	ERR9930693	
PolyA RNA-Seq Illumina	ERR10890695	
Genome assembly		
Assembly accession	GCA_946251885.1	
*Accession of alternate haplotype*	GCA_946251865.1	
Span (Mb)	803.3	
Number of contigs	185	
Contig N50 length (Mb)	9.4	
Number of scaffolds	50	
Scaffold N50 length (Mb)	27.4	
Longest scaffold (Mb)	39.3	
Genome annotation		
Number of protein-coding genes	19,901	
Number of gene transcripts	20,077	

* Assembly metric benchmarks are adapted from column VGP-2020 of “Table 1: Proposed standards and metrics for defining genome assembly quality” from ( Rhie *et al.*, 2021). ** BUSCO scores based on the lepidoptera_odb10 BUSCO set using v5.3.2. C = complete [S = single copy, D = duplicated], F = fragmented, M = missing, n = number of orthologues in comparison. A full set of BUSCO scores is available at https://blobtoolkit.genomehubs.org/view/ilCosPyra2.1/dataset/CAMIUC01/busco.

**Table 2.** Chromosomal pseudomolecules in the genome assembly of *Cosmia pyralina*, ilCosPyra2.

INSDC accession	Chromosome	Length (Mb)	GC%
OX276342.1	1	31.76	38.5
OX276343.1	2	31.43	38.0
OX276344.1	3	30.65	38.0
OX276345.1	4	30.01	38.5
OX276346.1	5	29.85	38.0
OX276347.1	6	29.17	38.5
OX276348.1	7	29.05	38.0
OX276349.1	8	29.02	38.0
OX276350.1	9	28.94	38.5
OX276351.1	10	28.51	38.0
OX276352.1	11	28.33	38.5
OX276353.1	12	27.47	38.0
OX276354.1	13	27.37	38.0
OX276355.1	14	26.93	38.0
OX276356.1	15	26.92	38.5
OX276357.1	16	26.81	38.5
OX276358.1	17	26.4	38.0
OX276359.1	18	26.1	38.0
OX276360.1	19	25.94	38.5
OX276361.1	20	25.37	38.5
OX276362.1	21	23.59	38.5
OX276363.1	22	23.24	38.0
OX276364.1	23	22.82	38.0
OX276365.1	24	22.36	38.5
OX276366.1	25	19.85	38.0
OX276367.1	26	18.73	38.0
OX276368.1	27	17.59	39.0
OX276369.1	28	17.15	39.0
OX276370.1	29	16.28	38.5
OX276371.1	30	15.44	38.5
OX276341.1	Z	39.28	38.5
OX276372.1	MT	0.02	19.5

The estimated Quality Value (QV) of the final assembly is 61.5 with *k*-mer completeness of 100%, and the assembly has a BUSCO v5.3.2 completeness of 99.0% (single = 98.1%, duplicated = 0.9%), using the lepidoptera_odb10 reference set ( *n* = 5,286).

Metadata for specimens, spectral estimates, sequencing runs, contaminants and pre-curation assembly statistics can be found at https://links.tol.sanger.ac.uk/species/987909.

## Genome annotation report

The *Cosmia pyralina* genome assembly (GCA_946251885.1) was annotated using the Ensembl rapid annotation pipeline ( Table 1; https://rapid.ensembl.org/Cosmia_pyralina_GCA_946251885.1/Info/Index). The resulting annotation includes 20,077 transcribed mRNAs from 19,901 protein-coding genes.

## Methods

### Sample acquisition and nucleic acid extraction

A male *Cosmia pyralina* (specimen ID Ox001826, individual ilCosPyra2) was collected in a light trap from Wytham Woods, Oxfordshire (biological vice-county Berkshire), UK (latitude 51.77, longitude –1.34) on 2021-07-24. The specimen was collected and identified by Douglas Boyes (University of Oxford) and preserved on dry ice.

DNA was extracted at the Tree of Life laboratory, Wellcome Sanger Institute (WSI). The ilCosPyra2 sample was weighed and dissected on dry ice with tissue set aside for Hi-C sequencing. Head and thorax tissue was disrupted using a Nippi Powermasher fitted with a BioMasher pestle. High molecular weight (HMW) DNA was extracted using the Qiagen MagAttract HMW DNA extraction kit. HMW DNA was sheared into an average fragment size of 12–20 kb in a Megaruptor 3 system with speed setting 30. Sheared DNA was purified by solid-phase reversible immobilisation using AMPure PB beads with a 1.8X ratio of beads to sample to remove the shorter fragments and concentrate the DNA sample. The concentration of the sheared and purified DNA was assessed using a Nanodrop spectrophotometer and Qubit Fluorometer and Qubit dsDNA High Sensitivity Assay kit. Fragment size distribution was evaluated by running the sample on the FemtoPulse system.

RNA was extracted from abdomen tissue of ilCosPyra2 in the Tree of Life Laboratory at the WSI using TRIzol, according to the manufacturer’s instructions. RNA was then eluted in 50 μl RNAse-free water and its concentration assessed using a Nanodrop spectrophotometer and Qubit Fluorometer using the Qubit RNA Broad-Range (BR) Assay kit. Analysis of the integrity of the RNA was done using Agilent RNA 6000 Pico Kit and Eukaryotic Total RNA assay.

### Sequencing

Pacific Biosciences HiFi circular consensus DNA sequencing libraries were constructed according to the manufacturers’ instructions. Poly(A) RNA-Seq libraries were constructed using the NEB Ultra II RNA Library Prep kit. DNA and RNA sequencing was performed by the Scientific Operations core at the WSI on Pacific Biosciences SEQUEL II (HiFi) and Illumina NovaSeq 6000 (RNA-Seq) instruments. Hi-C data were also generated from remaining head and thorax tissue of ilCosPyra2 using the Arima2 kit and sequenced on the Illumina NovaSeq 6000 instrument.

### Genome assembly, curation and evaluation

Assembly was carried out with Hifiasm ( Cheng *et al.*, 2021) and haplotypic duplication was identified and removed with purge_dups ( Guan *et al.*, 2020). The assembly was then scaffolded with Hi-C data ( Rao *et al.*, 2014) using YaHS ( Zhou *et al.*, 2023). The assembly was checked for contamination and corrected as described previously ( Howe *et al.*, 2021). Manual curation was performed using HiGlass ( Kerpedjiev *et al.*, 2018) and Pretext ( Harry, 2022). The mitochondrial genome was assembled using MitoHiFi ( Uliano-Silva *et al.*, 2022), which runs MitoFinder ( Allio *et al.*, 2020) or MITOS ( Bernt *et al.*, 2013) and uses these annotations to select the final mitochondrial contig and to ensure the general quality of the sequence.

A Hi-C map for the final assembly was produced using bwa-mem2 ( Vasimuddin *et al.*, 2019) in the Cooler file format ( Abdennur & Mirny, 2020). To assess the assembly metrics, the *k*-mer completeness and QV consensus quality values were calculated in Merqury ( Rhie *et al.*, 2020). This work was done using Nextflow ( Di Tommaso *et al.*, 2017) DSL2 pipelines “sanger-tol/readmapping” ( Surana *et al.*, 2023a) and “sanger-tol/genomenote” ( Surana *et al.*, 2023b). The genome was analysed within the BlobToolKit environment ( Challis *et al.*, 2020) and BUSCO scores ( Manni *et al.*, 2021; Simão *et al.*, 2015) were calculated.

Table 3 contains a list of relevant software tool versions and sources.

**Table 3.** Software tools: versions and sources.

Software tool	Version	Source
BlobToolKit	4.0.7	https://github.com/blobtoolkit/blobtoolkit
BUSCO	5.3.2	https://gitlab.com/ezlab/busco
Hifiasm	0.16.1-r375	https://github.com/chhylp123/hifiasm
HiGlass	1.11.6	https://github.com/higlass/higlass
Merqury	MerquryFK	https://github.com/thegenemyers/MERQURY.FK
MitoHiFi	2	https://github.com/marcelauliano/MitoHiFi
PretextView	0.2	https://github.com/wtsi-hpag/PretextView
purge_dups	1.2.3	https://github.com/dfguan/purge_dups
sanger-tol/genomenote	v1.0	https://github.com/sanger-tol/genomenote
sanger-tol/readmapping	1.1.0	https://github.com/sanger-tol/readmapping/tree/1.1.0
YaHS	yahs-1.1.91eebc2	https://github.com/c-zhou/yahs

### Genome annotation

The BRAKER2 pipeline ( Brůna *et al.*, 2021) was used in the default protein mode to generate annotation for the *Cosmia pyralina* assembly (GCA_946251885.1) in Ensembl Rapid Release.

### Wellcome Sanger Institute – Legal and Governance

The materials that have contributed to this genome note have been supplied by a Darwin Tree of Life Partner. The submission of materials by a Darwin Tree of Life Partner is subject to the **‘Darwin Tree of Life Project Sampling Code of Practice’**, which can be found in full on the Darwin Tree of Life website here. By agreeing with and signing up to the Sampling Code of Practice, the Darwin Tree of Life Partner agrees they will meet the legal and ethical requirements and standards set out within this document in respect of all samples acquired for, and supplied to, the Darwin Tree of Life Project. 

Further, the Wellcome Sanger Institute employs a process whereby due diligence is carried out proportionate to the nature of the materials themselves, and the circumstances under which they have been/are to be collected and provided for use. The purpose of this is to address and mitigate any potential legal and/or ethical implications of receipt and use of the materials as part of the research project, and to ensure that in doing so we align with best practice wherever possible. The overarching areas of consideration are:

• Ethical review of provenance and sourcing of the material

• Legality of collection, transfer and use (national and international) 

Each transfer of samples is further undertaken according to a Research Collaboration Agreement or Material Transfer Agreement entered into by the Darwin Tree of Life Partner, Genome Research Limited (operating as the Wellcome Sanger Institute), and in some circumstances other Darwin Tree of Life collaborators.

## Data Availability

European Nucleotide Archive: *Cosmia pyralina*. Accession number PRJEB54090; https://identifiers.org/ena.embl/PRJEB54090. ( Wellcome Sanger Institute, 2022) The genome sequence is released openly for reuse. The *Cosmia pyralina* genome sequencing initiative is part of the Darwin Tree of Life (DToL) project. All raw sequence data and the assembly have been deposited in INSDC databases. Raw data and assembly accession identifiers are reported in Table 1.

## Author information

Members of the University of Oxford and Wytham Woods Genome Acquisition Lab are listed here: https://doi.org/10.5281/zenodo.4789928.

Members of the Darwin Tree of Life Barcoding collective are listed here: https://doi.org/10.5281/zenodo.4893703.

Members of the Wellcome Sanger Institute Tree of Life programme are listed here: https://doi.org/10.5281/zenodo.4783585.

Members of Wellcome Sanger Institute Scientific Operations: DNA Pipelines collective are listed here: https://doi.org/10.5281/zenodo.4790455.

Members of the Tree of Life Core Informatics collective are listed here: https://doi.org/10.5281/zenodo.5013541.

Members of the Darwin Tree of Life Consortium are listed here: https://doi.org/10.5281/zenodo.4783558.
