# Supplementary material for: The genome sequence of the Lunar-spotted Pinion, Cosmia pyralina (Denis & Schiffermüller, 1775)
Source: Wellcome Open Res. 2023 Nov 23;8:545. [Version 1] doi: 10.12688/wellcomeopenres.20148.1 (PMC11408916; doi:10.12688/wellcomeopenres.20148.1)
Supplement: Peer review history

# Reviewer report: Reviewer response for version 1

**DOI:** 10.21956/wellcomeopenres.22310.r82491

**Published:** 2024-09-17

**Authors:** Jose Martinez

**Affiliations:** 1 University of Florida, Florida, USA

Boyes and Januszczak present a highly complete and meticulously assembled genome of a male Lunar-spotted Pinion, *Cosmia pyralina* (Denis & Schiffermüller, 1775). The assembly, distinguished by an outstanding BUSCO score, is arranged into 31 chromosomal pseudomolecules, including the fully recovered Z sex chromosome. The comprehensive quality of this genomic resource reflects the use of cutting-edge sequencing technologies and rigorous assembly methodologies. This high-quality dataset provides an invaluable tool for evolutionary studies, offering deep insights into the species’ genetic makeup and laying a strong foundation for future research on systematics, population genomics, and the broader evolutionary history of Lepidoptera.

 The development of this dataset is founded on a clear, well-structured rationale, supported by concise and accessible descriptions that enhance its usability. The carefully selected protocols and methodologies are meticulously designed to ensure both accuracy and reproducibility, adhering to the highest standards of scientific rigor. The dataset is highly standardized, allowing for seamless integration into various research frameworks. Accompanied by comprehensive and detailed illustrations, it provides a visually rich resource that enhances understanding and facilitates data interpretation. Furthermore, its accessibility ensures that it can be readily utilized by researchers across multiple disciplines, making it a versatile and invaluable asset for advancing knowledge in numerous scientific fields.

 Finally, the authors tackle a critical issue in natural ecosystems: "insect declines." The species in question, along with others in its genus, appears to be disappearing from Britain, likely due to the spread of Dutch elm disease. This genomic study provides valuable insights into the biology of this species and could play a pivotal role in its conservation.

Are sufficient details of methods and materials provided to allow replication by others?

Yes

Is the rationale for creating the dataset(s) clearly described?

Yes

Are the datasets clearly presented in a useable and accessible format?

Yes

Are the protocols appropriate and is the work technically sound?

Yes

Reviewer Expertise:

Evolutionary Biology, Ecology, IPM, Integrative Biology, Entomology, Genomics

I confirm that I have read this submission and believe that I have an appropriate level of expertise to confirm that it is of an acceptable scientific standard.

# Reviewer report: Reviewer response for version 1

**DOI:** 10.21956/wellcomeopenres.22310.r91944

**Published:** 2024-08-15

**Authors:** Ningning Wu, Shaolong Qiu

**Affiliations:** 1 College of Plant Protection, China Agricultural University College of Plant Protection, Beijing, Beijing, China; 2 China Agricultural University Education Foundation, Beijing, Beijing, China

In this study, the authors assembled the genome of the forestry insect *Cosmia pyralina*. They utilized a single adult male specimen and integrated PacBio HiFi sequencing, Hi-C sequencing, and transcriptome sequencing to produce a chromosome-level genome and achieve comprehensive annotation of protein-coding genes. The high quality of the assembly is demonstrated by the favorable QV and BUSCO values.

 The rationale for creating the dataset(s) is clearly described. However, while the protocols used are appropriate and the work is technically sound to some extent, there are areas where improvements could be made. The paper notes that the transcriptome sequencing was performed on the insect's abdomen. It might be worth considering whether gene annotation based solely on this abdominal transcriptome could fully capture all coding genes, as additional transcriptome data from other tissues might offer a more comprehensive annotation.

 Furthermore, the authors did not address the annotation of repetitive sequences in the genome, which could be an important aspect to include in the analysis. The methods and materials are detailed enough to allow for replication by others, and the datasets themselves are presented in a clear, usable, and accessible format.

Are sufficient details of methods and materials provided to allow replication by others?

Yes

Is the rationale for creating the dataset(s) clearly described?

Yes

Are the datasets clearly presented in a useable and accessible format?

Yes

Are the protocols appropriate and is the work technically sound?

Partly

Reviewer Expertise:

Comparative genomics; Population genetics; Toxicology

We confirm that we have read this submission and believe that we have an appropriate level of expertise to confirm that it is of an acceptable scientific standard.

## References

[1] : A high-quality genome assembly of the waterlily aphid Rhopalosiphum nymphaeae. *Sci Data* .2024;11(1) : 10.1038/s41597-024-03043-3 194 10.1038/s41597-024-03043-3 38351256 PMC10864314
